# The Impact of Induction Regimes on Immune Responses in Patients with Multiple Myeloma

**DOI:** 10.3390/cancers13164090

**Published:** 2021-08-13

**Authors:** Michael A. Firer, Michael Y. Shapira, Galia Luboshits

**Affiliations:** 1 Department Chemical Engineering, Ariel University, Ariel 40700, Israel; Galialu@ariel.ac.il; 2 Adelson School of Medicine, Ariel University, Ariel 40700, Israel; 3 Ariel Center for Applied Cancer Research, Ariel University, Ariel 40700, Israel; 4 The Hematology Institute, Assuta Medical Center, Tel Aviv 6971028, Israel; michaelshapi@assuta.co.il

**Keywords:** multiple myeloma, induction therapy, bortezomib, lenalidomide, melphalan, dexamethasone

## Abstract

**Simple Summary:**

Multiple myeloma remains an essentially incurable blood cancer. New patients with multiple myeloma are often treated with induction chemotherapy, stem cell transplantation, and then maintenance chemotherapy. As the immune system of the patient is a critical element in controlling the growth of tumor cells, this review aims to summarize what is known regarding the effects on the immune system of the commonly employed drugs used in the induction chemotherapy phase of the treatment—melphalan, dexamethasone, lenalidomide, and bortezomib. Understanding the effects of each drug on the immune system could lead to devising rational combinations of these drugs, which may lead to longer survival of patients with this cancer.

**Abstract:**

Current standard frontline therapy for newly diagnosed patients with multiple myeloma (NDMM) involves induction therapy, autologous stem cell transplantation (ASCT), and maintenance therapy. Major efforts are underway to understand the biological and the clinical impacts of each stage of the treatment protocols on overall survival statistics. The most routinely used drugs in the pre-ASCT “induction” regime have different mechanisms of action and are employed either as monotherapies or in various combinations. Aside from their direct effects on cancer cell mortality, these drugs are also known to have varying effects on immune cell functionality. The question remains as to how induction therapy impacts post-ASCT immune reconstitution and anti-tumor immune responses. This review provides an update on the known immune effects of melphalan, dexamethasone, lenalidomide, and bortezomib commonly used in the induction phase of MM therapy. By analyzing the actions of each individual drug on the immune system, we suggest it might be possible to leverage their effects to rationally devise more effective induction regimes. Given the genetic heterogeneity between myeloma patients, it may also be possible to identify subgroups of patients for whom particular induction drug combinations would be more appropriate.

## 1. Introduction

Multiple myeloma (MM) is a malignancy of plasma cells and the second most common hematological cancer. MM results from an antigen-experienced B cell clone that undergoes transformation, clonal evolution and expansion, and eventual proliferation in the bone marrow (BW) [1]. This process is mirrored by laboratory-defined, pre-malignant states known as monoclonal gammopathy of undefined significance (MGUS) and smoldering MM (SMM), which are eventually replaced by a state of active, clinical disease. The progression rates from MGUS and SMM to active MM are approximately 1% and 10% per year, respectively [2]. These transformed clones owe their survival and proliferation to complex interactions with a spectrum of cells within the BM microenvironment, which include secretion of cytokines such as IL-6 by stromal cells and dampening of immune cell activity [3] characterized by progressive immune suppression and exhaustion [4].

Current standard frontline therapy for eligible MM patients involving induction therapy, autologous stem cell transplantation (ASCT), and maintenance therapy improves overall survival [5,6,7,8] (https://www.nccn.org/professionals/physician_gls/pdf/myeloma.pdf accessed on 1 July 2021) and helped elevate the 5 year survival rate to approximately 50% [9]. Nonetheless, essentially all patients eventually relapse and die within 7–10 years [10]. Several studies demonstrated that patients characterized as long-term survivors appear to develop a particular immune cell profile, and major efforts are underway to understand the immunological and [11,12] the clinical impacts of each stage of the treatment protocols on overall survival statistics. One important component of this process is the pre-ASCT “induction” regime, whose primary aim is to significantly reduce tumor burden, thus improving the chances that the immune system will eliminate any remaining tumor cells, particularly following hematopoietic reconstitution post-ASCT and achieving a laboratory status of minimal residual disease (MRD). Support for this approach comes from studies showing the induction of antigen-specific CD8+ memory T cells in mice with controlled disease [7] and from the recent PETHEMA/GEM2012MENOS65 trial, which demonstrated longer progression free survival (PFS) rates in those patients who achieved MRD negativity following this treatment process [13]. Those results are backed up by previous studies [14,15] suggesting that the composition of the induction regimen impacts the chance of achieving and maintaining MRD negativity.

The choice of drug(s) used in the pre-ASCT induction phase has evolved over the years and now includes those with different mechanisms of action, including alkylating agents (e.g., melphalan), corticosteroids (e.g., dexamethasone), immunomodulatory drugs (IMIDs) (e.g., lenalidomide), proteasome inhibitors (PIs) (e.g., bortezomib), and monoclonal antibodies (mAbs) (e.g., anti-CD138), either as monotherapies or in various combinations. However, aside from their direct effects on cancer cell mortality, these drugs can also influence the anti-tumor functionality of the total immune system by stimulating and/or suppressing different immune cells. Moreover, as emphasized recently by Minnie and Hill [8], the kinetics and the extent of post-ASCT immune reconstitution, including analysis of the immune cell subsets responsible for maintaining a robust anti-tumor response, may help to explain the long-term PFS and overall survival (OS) of some patients with MM who receive this therapy. This observation raises the important question as to how different induction regime drugs impact post-ASCT immune reconstitution and subsequent anti-tumor immune responses. This issue is further worthy of consideration given the dependence on survival and proliferation of MM cells in their intricate relationship with various components of the BM microenvironment and their ability to influence the functionality of infiltrating immune cells [16,17]. To begin answering this question, this review provides an update on the known immune effects of the most prominent drugs currently used in the pre-ASCT induction phase, focusing on melphalan, dexamethasone, lenalidomide, and bortezomib in the treatment of MM. By analyzing the action of each individual induction drug, especially on the immune system, it might be possible to leverage their effects to rationally devise more effective induction regimes. Given the genetic heterogeneity between myeloma patients, it may also be possible to identify subgroups of patients for whom particular induction drug combinations would be more appropriate.

## 2. Immune Efforts of Individual Induction Regime Drugs

### 2.1. Melphalan

**Figure 1:**
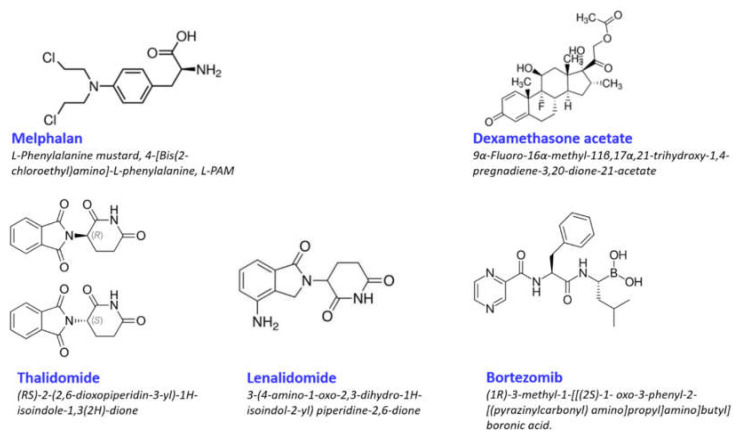
Chemical structures of drugs commonly used in the induction phase for the treatment of patients with multiple myeloma. Thalidomide is included only for comparison to its derivative lenalidomide.

Melphalan (Mel) is the phenylalanine derivative of nitrogen mustard and, as such, is a member of the nitrogen mustard group of alkylating drugs (Figure 1). Its cytotoxic activity derives from its ability to induce inter- or intra-structural DNA-DNA as well as DNA–protein cross-links via its two chloroethyl groups, leading to DNA strand cleavage and disruption of DNA replication and transcription [18,19]. Although first introduced to the clinic in the mid-1980s, the use of Mel followed by ASCT has remained a recommended treatment of patients with newly diagnosed MM <65 years of age who are eligible for transplantation, even though Mel can lead to side effects in gastrointestinal, cardiac, and hepatic systems [19,20].

Aside from its direct cytotoxic activity, Mel was shown in a number of studies to have several effects on immune cells (Table 1). Using an A20 murine model of B-cell lymphoma, Lu et al. [21] demonstrated that Mel resulted in transient depletion of monocytes, granulocytes/neutrophils, and conventional dendritic cells (DCs), whereas there were only modest fluctuations in macrophages and plasmacytoid DCs (pDCs). They further showed that melphalan treatment induced a transient burst of inflammatory cytokines and chemokines that recruited both innate immune cells and activated T cells. Treatment of tumor bearing mice with Mel led to antigen expression on the surface of the A20 cells, uptake of antigens and activation of DCs, and priming of tumor-infiltrating CD8+ T cells, effector memory cells (CD25, CD44, CD621L), and granzyme B. Finally, adoptive transfer of antigen-specific CD4+ T cells into melphalan-treated hosts resulted in their clonal expansion of the T cells, downregulation of PD-1 and Foxp3, upregulation of CD40L, and increased cytokine production, which translated into tumor regression and marked prolongation of mouse survival. These data indicate that Mel induces immune-induced cell death (ICD), a concept related to the ability of chemotherapeutics to cause stress or damage in tumor cells, leading to their expression of damage-associated molecular patterns (DAMPs) recognizable by immune cells [22].

While these immune-based anti-tumor consequences of Mel induction therapy are promising, there are also paradoxical situations which complicate its use. For example, CD28 is classically known as an essential ligand expressed on CD4+ T cells undergoing activation by B7(CD80/CD86) expressing antigen-presenting cells (APCs) such as DCs. Although Mel enhances B7 expression on tumor cells, which leads to engagement of tumor reactive T cells, it has been known for some time that MM cells can also express CD28 and that the level of expression correlates with disease progression and poor prognosis [23]. Indeed, further studies showed that interaction between CD28+MM cells and B7+DCs led to decreased tumor cell apoptosis [24] and production of pro-MM survival and immunosuppressive signals such as IL-6 and indolamine-2,3-dioxygenase (IDO), respectively [44]. In the context of achieving MRD negativity post-ASCT [13], such anomalies in the immune effects of Mel require further study so as to learn how best to use this drug in the pre-ASCT induction setting.

### 2.2. Dexamethasone

Dexamethasone (Dex) is a glucocorticosteroid (Figure 1) and exhibits pleotropic effects [45] on cells. Corticosteroids such as Dex are commonly used in cancer chemotherapy to relieve dyspnea, bowel obstruction, spinal cord compression, and pain from bone metastases and neurological symptoms [46]. Dex is the most commonly used synthetic glucocorticoid, as it has a longer half-life and a higher binding affinity for glucocorticoid receptors [47]. Notably, however, studies showed that extended use of Dex can lead to drug resistance in several types of cancer cells by downregulation of glucocorticoid receptors [48] or enhanced adhesion to the extracellular matrix by overexpression of β1, α4, and α5 integrins [49].

Corticosteroids are also often used to control immune-related adverse events (irAEs) such as autoimmune or allergic reactions that can emerge as a consequence of immune checkpoint blockade therapies [45,50], although the mechanism(s) by which these molecules impact cellular immunity is not well understood [25]. Clearly, corticosteroids do have potent regulatory effects on inflammation and T cell activity (Table 1). In a mouse model of glioma, Dex upregulated CTLA-4 mRNA and protein in CD4+ and CD8+ T cells, especially naïve T cells [25], although these effects were not always seen in healthy human peripheral blood mononuclear cells (PBMCs) [51]. Other reported effects of Dex on human T cell activity include a reduction in the kinetics of cell division of both CD4 and CD8 T cells, possibly by blocking the G_0_/G_1_ cell cycle transition [25], and reduction in production of important T cell effector cytokines such as IL-2, IFN-γ, and TNF-α [51]. In addition to its effects on immune cells from healthy individuals, Dex significantly reduced the in vitro anti-tumor reactivity of tumor-infiltrating lymphocytes (TILs) in samples from patients with stage IV melanoma, [26]. As would be expected, these effects are not target cell specific and would also be apparent outside of anti-tumor immunity. Indeed, premedication of tumor-bearing mice with Dex inhibited vaccine-dependent induction of serum cytokines and chemokines and reduced both the number and the activation of conventional DCs expressing vaccine encoded antigens [52], suggesting that induction therapy with Dex alone might impair off-target immune responses. In fact, protocols involving Dex are a risk factor for infection in MM patients undergoing induction therapy [27]. Nonetheless, other data suggest that the implications of these effects for tumor immunity should be made with caution, as a recent study by Aston et al., demonstrated that, while Dex treatment of mesothelioma-bearing mice resulted in significant decreases in peripheral blood CD4+, CD8+ T cells, T-regs, B cells, and NK cells, the tumor infiltration by these immune subsets was not affected. Similarly, T cell activation and proliferation or immune checkpoint receptor expression were not altered, suggesting that factors within the tumor microenvironment may protect these cells from Dex. It should be noted that studies on the immune effects of Dex alone in patients or mice are lacking. Still, in practice, Dex is not employed as a monotherapy in MM induction protocols but rather in combination with other drugs which might override or compensate for its negative effects on the immune system. One such example is a recent report of a phase 1/2 study by the Paiva group of RRMM patients in which addition of Dex to treatment with the anti-CD38 antibody isatuximab did not impede expansion of T cell clonality in responding patients and was associated with a higher overall response rate [53].

### 2.3. Lenalidomide

Lenalidomide (Revlamid^®^) (Rev) is a derivative of thalidomide, itself a synthetic derivative of glutamic acid (Figure 1), which was initially used in the late 1950s as a sedative and to reduce the effects of “morning sickness”. Tragically, the early 1960s saw reports of birth defects such as stunted limb development in babies born to mothers who used the drug, and it was subsequently withdrawn from the market. Thalidomide exists as an equal mixture of S- (−) and R- (+) enantiomers with the S form having teratogenic properties. Unfortunately, as the enantiomers spontaneously and rapidly interconvert under physiologic conditions, the isolation and the use of only the R enantiomer are impractical [54]. For more on the history of thalidomide and regulatory strategies later put in place to ensure withholding of its use during pregnancy, the reader is referred to comprehensive reviews [55,56].

Despite its teratogenicity, subsequent studies demonstrated that thalidomide also had anti-inflammatory, antiangiogenic, and immunomodulatory properties, prompting its testing as a therapeutic in the treatment of autoimmune disease and cancer [57]. To overcome the drug’s side effects, such as neuropathies and deep vein thrombosis, two classes of derivatives were developed. One class acts as selective cytokine inhibitors, while the other includes a series of compounds grouped under the somewhat confusing name “immunomodulatory drugs (IMiDs)” [58]. Rev, one of these IMiDs, is 2000 times more potent than thalidomide in inhibiting the production of the pro-inflammatory cytokines TNF-α [29], IL-1b, IL-6, and IL-12 production and in increasing IL-2 and interferon (IFN)-γ synthesis [28] (Table 1). Thalidomide and Rev were approved for clinical use in patients with MM in 2006 and before details of their mechanism(s) of action on the anti-MM immune response began to be highlighted. Nonetheless, their therapeutic efficacy, especially that of Rev, was clearly demonstrated in first line therapy for newly diagnosed MM patients whether or not they were eligible for ASCT as maintenance therapy in patients who received ASCT as well as in those patients with refractory/relapsed disease [28].

A wide range of studies demonstrate that Rev (as its parent molecule thalidomide) has both immunologically- and extra-immunologically-derived effects of MM cells. First, Rev is directly cytotoxic for MM cells via its effect on several intracellular pathways, including activation of apoptotic caspases and downregulation of Wnt/β-catenin signaling pathways [59,60,61]. Rev was found to bind cereblon (CRBN) [62], a protein substrate receptor of the CRL4 E3 ligase. One of the consequences of the CRBN–E3 ligase interaction is augmentation of the selective ubiquitination and degradation of two transcription factors, IKZF1 and IKZF3, and the subsequent loss of interferon regulatory factor 4 (IRF4) and MYC expression found to be essential for myeloma cell proliferation [63]. Indeed, the re-expression of IRF4 conferred relative IMiD resistance to the MM cell line MM.1S [64]. Second, Rev is involved in disrupting interactions between various components of the BM microenvironment that myeloma cells subvert to promote their survival and proliferation [65]. These include the production of IL-6 by BM stromal cells [66] and mononuclear cells [67], angiogenesis [68], and the complex interplay between myeloma cells, osteoblasts, and osteoclasts [69].

Third, Rev has myriad effects on immune cells, which complicates the derivation of a coherent mechanism of action. For example, there are conflicting reports of its effects on Tregs. Most but not all studies indicate that addition of Rev to cell cultures leads to proliferation of T cells with regulatory phenotypes of CD4+CD25+ and CD8+CD28−, in the latter case possibly by the production of IL-10 by DCs [67], although, at least for PBMCs cultured with IL-2, addition of Rev led to a 50% reduction in the number of CTLA-4+CD25^high^ CD4+ Tregs with decreased FOXP3 expression [31]. Notwithstanding, it is usually found that Rev augments anti-tumor immune activity, as reported by Chung et al., who showed that, at least for post ASCT lenalidomide maintenance therapy, Tregs declined as CD8+ T cells expanded during early lymphocyte recovery [30]. Rev was shown to synergize with checkpoint PD-1/PD-1L blockage to enhance anti–MM immune responses [70], and Rev can lead to increased IFN-γ production in antigen-specific T cells, even in the presence of CD8+CD28- Tregs [67], as well as in MM-derived CD8+ T cells stimulated with anti-CD3 and anti-CD28 antibodies [71]. Rev was reported to augment NK cell activity by several mechanisms such as increasing their production of IFN-γ and TNF-α [72], possibly by up-regulating the expression of the TNF-related apoptosis-inducing ligand (TRAIL) [72] and by enhancing the expression of NK-cell-activating receptor ligands such as MICA and PVR/CD155 in malignant plasma cells [28].

Together with the demonstration that IMiDs improve the quality of antigen-specific T cells induced or expanded by DCs [73], it is clear that Rev can augment broad and polyfunctional antigen-specific anti-MM cellular immune responses. Moreover, the complementary features of Rev, that is, its ability on the one hand to overcome protection of MM cells afforded by the BM microenvironment and on the other to promote anti-tumor immunity while reversing the effects of myeloma-induced immune suppression, have significant benefits for MM patients. Notwithstanding the clinical benefits, further studies are needed to more accurately define the mechanisms by which IMiDs perform these diverse actions.

In addition, there are conflicting reports on the immune effects of long-term maintenance therapy with Rev in MM patients. For example, Bissot reported that maintenance Rev polarized the peripheral blood immune response to an chronic inflammatory Th1 phenotype with increased production of IFN-γ and TNF-α by CD4+ T cells [74], while Lullo reported that reduced production of pro-inflammatory cytokines (IL-17, IL-22, IL-6, TNF-α) in the bone marrow correlated with Rev treatment and more favorable clinical outcomes [75]. A study by Fostier further confounds this topic. They reported that maintenance Rev had both immunostimulatory and inhibitory effects. On the one hand, they found an increase in naïve and memory CD8^+^ T cells but reduced numbers of terminal effector CD8^+^ T cells, higher expression of co-stimulatory molecules on resting T cells but also of inhibitory checkpoint molecules, and increased numbers of Tregs with a phenotype associated with strong suppressive capacity [76]. These conflicting results may be attributed to differences in the clinical backgrounds of the cohorts studied and their immune profiles following different induction therapies, as discussed below. Finally, details are lacking on the influence of Rev induction therapy on the immune status of MM patients prior to ASCT and following reconstitution.

### 2.4. Bortezomib

Bortezomib (Velcade^®^) (Vel) is a small molecule that interferes with the proteolytic activity of the 26S proteasome, a cytosolic protein complex that executes the ubiquitin-directed degradation of proteins that are either incorrectly folded, are no longer required, or which control crucial cellular processes, such as the rate of protein production. Since many of these processes are defective in cancer cells, the potential of proteasome inhibitors as therapeutic agents was already acknowledged in the mid-1990s [77]. An early target of this strategy was to inhibit the activation of the nuclear factor kappa light chain enhancer of the activated B cells (NF-κB) family of transcription factors. NF-κB regulates the expression of genes important in a number of cellular processes, including DNA replication, cytokine production, inflammation, immunity to infection, and cell survival [78,79]. By blocking degradation of the NFκB inhibitor IκB, nuclear translocation of NF-κB could be prevented [80]. This rationale led to the development of Vel, originally known as PS-341, a first generation dipeptidyl boronic acid (Figure 1) developed as a derivative of molecules related to calpain inhibitor I [81]. Vel inhibits chymotrypsin-like, trypsin-like, and glutamyl peptide hydrolyzing activities within the 20S core of the proteasome, leading to apoptosis of several types of cancer cells [82]. Vel-related apoptosis might be induced by several mechanisms. In cervical cancer cells, Vel induced cell cycle arrest, which led to mitochondrial-independent apoptosis, but the upregulation of the molecular chaperone BiP and the cell stress marker ATF3 led the study’s authors to conclude that induction of the unfolded protein response (UPR) was the main cause of apoptosis [83]. In contrast, treatment of melanoma cells with Vel led to apoptosis via mitochondrial dysregulation, activation of caspases 3 and 9, as well as autophagy [84]. More recently, analysis of global gene and miRNA expression in human neuroblastoma cells treated with Vel [85] revealed changes in expression of over 1000 genes and 89 miRNAs, including significant upregulation of genes engaged in apoptosis, such as clusterin (CLU), heme oxygenase (decycling) 1 (HMOX1), caspase 7, and DNA-damage-inducible transcript 3 (DDIT3). In line with these results, Stuhler and Nekova found that Vel decreased the expression in MM cells of Dicer1, a major enzymatic component of the processing machinery of miRNAs such as miR-128 [86]. Several additional lines of evidence suggested that Vel may be particularly effective in MM, not the least of which were the findings of increased NF-κB expression in the BM and in MM cells which could be associated with enhanced tumor cell survival [81]. The FDA granted accelerated approval for Vel in May 2003 for use as a single agent in the treatment of patients with MM after two prior therapies and progressing with their most recent therapy. For more details on the clinicals trials and the review process that led to the approval, the reader is referred to Kane et al., 2003 [87].

As is discussed later, many clinical studies validated the use of combination induction therapy, including the combination of Rev and Vel. Intriguingly, and as mentioned by Wang et al. [88], the intracellular actions of Rev and Vel would, a priori, appear incompatible. As described above, Rev leads to the proteasomal degradation of IKZF1 and IKZF3, two transcription factors essential for myeloma cell proliferation [63], whereas this process should be inhibited by Vel. Nonetheless, both drugs induce apoptosis in MM cells. It seems there remains much to be learned about the pharmacological interaction of these drugs. As for the previous compounds, there remains confusion as to the mechanism(s) by which Vel affects immune activity, with some studies describing augmentation of anti-tumor immunity, while others report the opposite [34]. Early examples of this discrepancy are in vitro results showing that Vel downregulated expression of MHC class I molecules on MM cell lines and patient MM cells while increasing the expression of NK recognition molecules such as KIR-L, which might explain the enhanced sensitivity of MM cells to NK cell-mediated lysis [32]; however, a latter study showed that Vel abrogated NK activity [33]. Similarly, conflicting results were reported on the negative [41,89] and the positive [42] effects of Vel on DC activity, although more recent studies by Gulla et al., in the 5TGM1 murine model showed that treatment with Vel led to ICD by increased expression of CD86/CD83 in immature DCs and their phagocytosis of MM cells and by activating the cGAS/STING innate immune response signaling pathway [90,91]. pDCs, a TNFα-producing subset of DCs, were found to be very susceptible to the action of Vel [34], although the role of tumor infiltrating pDCs in immunosuppression or promotion of tumor progression remains unresolved [92]. These inconsistencies are probably due to differences in experimental design, protocols, and source of cells. In any case, clinical experience clearly validates the beneficial effects of Vel therapy in MM patients, reiterating the dichotomy between experimental culture conditions which usually include higher, non-pharmacological doses and continuous mode of exposure with those encountered under in vivo physiological conditions [93].

The effects of Vel of lymphocytes were also investigated (Table 1). While studying the terminal differentiation of B lymphocytes into plasma cells, Cascio and colleagues found that Vel decreased polyclonal antibody responses to both T independent and dependent antigens and increased apoptosis of B cells [37]. These results were later confirmed independently [38]. Coincidentally, the effects of Vel on activated B cells and dividing plasma cells provide further insight into the observed efficacy of the drug in MM as compared to solid tumors [94] and led to the testing of Vel in related settings, such as antibody-mediated autoimmune disorders [95,96] and transplant rejection [97]. However, the results do raise the question as to the drug’s effects on overall humoral immune responses in treated patients. One study in renal transplant patients showed that, while Vel reduced the levels of donor specific anti-HLA antibodies, tetanus toxoid and measles IgG levels remained surprisingly unchanged and above the level of protection, even after 1 year post-treatment [98], while Celotto and co-workers found that IgG antibodies to common viral and vaccine antigens were not affected and were even sometimes enhanced in MM patients receiving Vel [99]. These data suggest that Vel does not lead to suppression of antibody responses to recall antigens which depend on memory B cells with slow turnover.

The sensitivity of T cell subsets to Vel appears to be dose and tumor type dependent. In solid tumor mouse models, Shanker et al., reported that low dose Vel (LD-Vel) had no significant effect either on total leukocyte counts or on the levels of CD4+ or CD8+ T cells in either control or tumor-draining lymph nodes, spleen, or tumor-infiltrating lymphocytes, whereas a high-dose regiment led to leukopenia and an increased number of lung metastases [100]. In similar models, LD-Vel was shown to augment activation signals in CD8+ T cells such as PI3K/Akt/STAT5 pathways and to significantly increase production of the immunostimulatory cytokines IL-2, IL-12, and IL-15 while decreasing the levels of tumor-promoting cytokines IL-1β and VEGF [101]. These effects on T cells may be related to direct activation of signaling pathways, as treatment of tumor bearing mice with Vel resulted in upregulation in activated CD8+ T cells of Notch pathway components and enhanced IFN-γ secretion and expression of the effector molecules perforin and granzyme B [35]. Alternatively, or in addition, the upregulation may be due to increased expression of miR-155, a key cellular miRNA known to be involved in T cell function [102]. Several studies also demonstrated a composite effect of Vel on both T- and NK cell activity. For example, in an in vitro system, the combination of a death receptor agonist (anti-TRAIL-R1) and low dose Vel led to enhanced anti-MM cytotoxicity of both CD8+ T cells and NK cells [36].

Vel was shown to suppress the proliferation of Th17 cells which, while potentially useful in the treatment of autoimmune disease [103], is difficult to interpret for cancer therapy. On the one hand, Th17 cells can induce immunosuppression and increase tumor angiogenesis, but they also mediate antitumor immune responses through recruitment of immune cells into tumors, stimulating effector CD8+ T cells [39]. Similarly, the role of Tregs in MM remains unclear. While they are critical for the control of potential autoreactive T cell, their roles in cancer promotion or rejection can be paradoxical [104], as can be seen from the limited literature on their role in MM. For example, it is not clear if or how Vel affects the elevated percentages of Tregs seen in patients with MM [40,43]. However, this may depend on the source of the Tregs and the mode of Vel therapy. Data from Blanco and colleagues show that “naturally occurring”, thymus-generated CD4^+^CD25^+^ Tregs are resistant to Vel, whereas long-term exposure of peripheral CD4^+^ T cells to the drug promotes the expansion a Treg subset that significantly inhibits proliferation, IFN-γ production, and CD40L expression among stimulated effector T cells [105].

As noted previously for the other compounds discussed in this review, there remains a lack of detailed in vivo studies on the immune profile of either MM patients or in MM animal models following mono-induction therapy with Vel.

## 3. Combination Induction Regimes

The previous sections of this review aimed to describe the individual effects on the immune system of drugs commonly used in induction therapy for MM. According to the latest EHA-ESMO Clinical Practice Guidelines [106] and NCCN Clinical Practice Guidelines in Oncology (https://emedicine.medscape.com/article/204369-guidelines#g2 accessed on: 12 July 2021, high dose Mel (200 mg/m^2^) remains the standard conditioning regimen before ASCT for newly diagnosed MM (NDMM) patients, although a combination of a protease inhibitor and IMiD and a steroid is also recommended. Indeed, aside from Mel, the trend over recent years has been to combine several of these drugs together so as to either synergize the properties of the drugs or to counter certain negative effects of one of them. Numerous combinations were tested, the most efficacious of which are three-drug composites such as Vel/Rev/Dex, Vel/Dex/Cyclophosphamide, and Carfilzomib/Rev/Dex [107,108], although the addition of monoclonal antibodies such as daratumumab (anti-CD38) is also garnering interest [109]. These drug combinations were devised empirically [107] rather than by rational design, and their clinical efficacies both prior to and following ASCT were the subject of many clinical trials and publications. Unfortunately, their parallel effects on MM or immune cells have not often been reported.

Although Rev and Dex are a common drug combination in MM therapy [110,111], early studies showed that the NK-mediated killing of MM cells enhanced in the presence of Rev and CD4+ T cells was abrogated by the addition of Dex [112,113]. These results indicate that, at least from an immunological viewpoint, the combination of Rev and Dex would appear to be counter-productive, mirroring the pharmacological paradox of this drug mixture mentioned above. However, in practice, the experience seems different. In a study of patients with SMM, Praiva and colleagues analyzed the peripheral blood immune profiles of 13 patients following induction cycles Rev/Dex and again during maintenance with lenalidomide, finding no significant differences in the absolute numbers of total lymphocytes or in the ratio of the CD4+ and the CD8+ T cells [114]. More recently, paired blood samples were collected from 33 NDMM patients enrolled in the POLLUX trial [115] prior to and after 2 months treatment of lenalidomide (25 mg orally on days 1 to 21, six cycles) and dexamethasone (40 mg weekly). Deep immune profiling showed that this combination did not downregulate CD38 expression, the expansion in memory CD8+ T cells was not significant, nor was there any significant change in the proportions of other T cell subsets. Popadimitriou and colleagues [116] recently reported data on deep immunoprofiling for a series of patients, including 53 with NDMM who all received a combination induction regime of Vel/Rev/Dex (VRD). While the immune profile following VRD therapy was not specifically determined, those patients who went on to a clinical complete response (CR) could be immunologically distinguished at diagnosis by a BM compilation that included elevated total percentage of T cells, in particular CD27+ T cells, and decreased tumor-infiltrating macrophages. In peripheral blood, these patients had lower percentages of Tregs and a higher ratio of terminal effector/resting Tregs. Thus, immune profiling could be used to predict response to therapy. Lee et al., found that the value of immune profiling to predict clinical outcomes in RRMM patients receiving Rev/Dex was cycle dependent. For example, failure to achieve a very good partial response correlated with a decrease in CD8+ T cells and an increase in monocyte- myeloid-derived suppressor cell frequency after three cycles of treatment. Additionally, a high proportion of CD3+CD56+ natural killer T-like cells prior to Len/Dex treatment predicted a longer time to progression [117] Similarly, a multi-center Spanish Phase 3 trial (PETHEMA/GEM2012, #NCT01916252) tested the efficacy of six cycles of VRD induction in 458 patients with NDMM, who then received ASCT. The rates of a very good partial response or better increased with cycle number. Immune profiles in these patients were not reported [108]. In contrast, Ho et al., found no correlation between progression free survival or overall survival and the type IMiD used in therapy [12]. The conflicting data on the immune effects of Rev/Dex combination may reflect the difference between in vitro and clinical studies or different patient populations and highlights the need to carefully design animal model experiments that can lead toward a better understanding of the effects of induction drug combinations on the anti-myeloma response.

## 4. Conclusions

**Figure 2:**
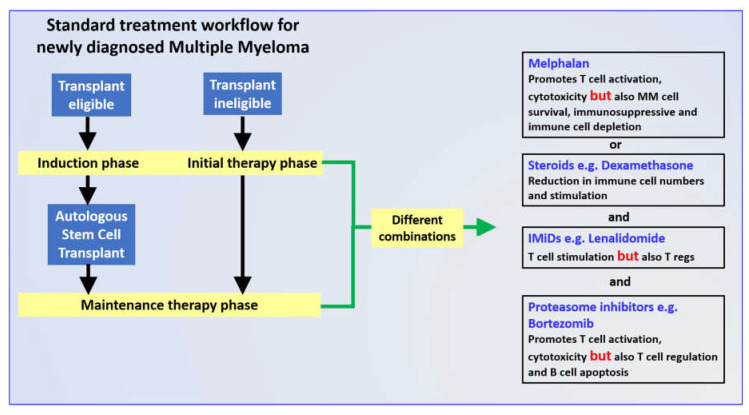
Unresolved issues regarding the impact of commonly used drugs on the immune responses of patients with multiple myeloma. Some drugs can have both immunostimulatory and regulatory effects. Their combination may impact the ability of the patient to mount effective, long-term anti-tumor immunity. Further research in clarifying these effects, especially with in vivo systems, may lead to effective drug therapy and improved patient survival.

Numerous studies demonstrated the superior clinical efficacy of induction therapy followed by ASCT and maintenance therapy for eligible patients with NDMM (Figure 2). Given the importance of both the induction phase in the treatment protocol [107,118] and the role of the immune response [119,120] in the overall survival of MM patients, we described in detail the impact of individual induction drugs on both the innate and the adaptive immune responses, noting the conflicting results often reported. Many of these studies used different in vitro experimental systems, complicating a comparison of the data and the accurate definition of the compound’s mechanism(s) of action. Notably, there is a surprising lack of data from in vivo systems for both animal models and patients tracking the impact of the drugs reviewed here on immune profiles. This is particularly the case for drug combinations, of which the use of some (Vel/Rev) appear at first sight to be contradictory. Facilitating these studies are recent efforts to use cell lines and patient cells to construct 3D organoids to reconstruct the bone marrow environment [121,122]. These systems are being used to study the pathogenesis of MM [123] and to develop new chemo- and immune therapies [124,125]. Given that MM remains an incurable disease, this information might allow a more rational assembly of induction therapy combinations that could be personalized and contribute to a more resilient anti-MM immune response and extended survival.

## Figures and Tables

**Table 1:** Main effects on immune parameters of pre-ASCT induction drugs used to treat patients with Multiple Myeloma.

Drug	Essential MOA	Effects on Innate Immune System	System	Ref	Effects on Adaptive Immune System	System	Ref
Melphalan (Mel)	Induction of DNA–DNA, DNA–protein cross-links leading to disruption of DNA replication and transcription; tumor cell death	Negative -Transient depletion of monocytes, granulocytes, and conventional DCs	AM	[21]	Positive -Transient burst of inflammatory cytokines;	AM	[21]
					-Priming of tumor-infiltrating activated, CD8+ T_EM_ cells	AM	[21]
					-Induction of ICD	AM	[21]
		-Transient burst of inflammatory cytokines	AM	[21]	-Proliferation of antigen-specific CD4+ T cells	AM	[21]
					-Enhances B7 expression of tumor cells and DCs	EV	[23]
					Negative CD28+MM/B7+DC interaction enhances MM cell survival	EV	[23,24]
Dexamethasone (Dex)	-Immunosuppression, particularly T cells				Negative -Reduction in T cell stimulation by upregulation of CTLA-4/PD-1 expression	IV	[25]
					-Reduction in cytokine production	IV	[25]
					-Reduction in peripheral blood CD4+, CD8+ T cells, T-regs, B cells, and NK cells	IV	[25]
					-Reduction in TIL activity vs. autologous tumor cells	IV	[26]
					-Risk factor for infection in MM patients	Cl	[27]
Lenalidomide (Revlamid^®^) (Rev)	-Immunomodulation, antiangiogenic	Positive -Enhances production of IFN-γ and TNF-α in NK cells	EV, Cl	[28]	Positive -Inhibition of pro-inflammatory cytokines TNF-α, IL-1b, -6 -12	IV	[29]
					-Increased production of IL-2, IFN-γ	EV, Cl	[28]
					-Increased IFN-γ production in CD8+ T cells	EV, Cl	[28]
					- Tregs decline as CD8+ T cells expand	EV, Cl	[30]
	-Binds cereblon leading to reduction in growth of MM cells				Negative May enhance production of Tregs	IV, EV	[31]
	-Inhibits production of IL-6 by BM stromal cells						
Bortezomib (Velcade^®^) (Vel)	-Inhibitor of proteasomal enzymes	Conflicting -Effect of NK activity	IV, EV	[32,33]	Positive Low-dose -Increase in CD8+T cell activation and production of IL-2,-12,-15	AM, IV	[34,35]
					-Increase in CD8+ T cell and NK cytotoxicity	IV	[36]
					Negative -Decreased polyclonal antibody production; increased B cell apoptosis	AM, IV	[37,38]
					Conflicting -Effect on Th17+ T cells	AM	[39,40]
	-blocks activation of NFκB	-Effect on DC activity	IV, EV	[41,42]	-Effect on Tregs	AM, Cl	[43]

AM—animal model; ASCT—autologous stem cell transplantation; BM—bone marrow; Cl—clinical; DC—dendritic cell; EV—ex vivo; ICD—immunogenic cell death; IV—in vitro; T_EM_—effector memory T cells; TIL—tumor infiltrating lymphocytes; Tregs—T regulatory cells.
